# In Addition to Poor Glycemic Control, a High Level of Irisin in the Plasma Portends Early Cognitive Deficits Clinically in Chinese Patients With Type 2 Diabetes Mellitus

**DOI:** 10.3389/fendo.2019.00634

**Published:** 2019-09-13

**Authors:** Hongyan Lin, Yang Yuan, Sai Tian, Jing Han, Rong Huang, Dan Guo, Jiaqi Wang, Ke An, Shaohua Wang

**Affiliations:** ^1^Department of Endocrinology, Affiliated Zhongda Hospital, Southeast University, Nanjing, China; ^2^School of Medicine, Southeast University, Nanjing, China

**Keywords:** irisin, mild cognitive impairment, executive function, diabetes mellitus, insulin resistance

## Abstract

**Background and Objectives:** Irisin plays an important role in the metabolism and homeostasis of energy balance, which is involved in cognitive impairment. This study aimed to investigate the role of irisin in mild cognitive impairment (MCI) among Chinese patients with type 2 diabetes mellitus (T2DM).

**Methods:** We recruited 133 Chinese patients with T2DM, and divided them according to the Montreal Cognitive Assessment score. Demographic data were collected and the level of irisin in the plasma was determined. In addition, the results of neuropsychological testing were examined. The concentration of irisin in the plasma was measured using an enzyme immunoassay.

**Results:** A total of 59 patients were diagnosed with MCI and 74 patients were included as healthy-cognition controls. The level of irisin in the plasma (*p* = 0.043) and homeostasis model of assessment for insulin resistance (*p* = 0.032) in diabetic patients with MCI were higher than those observed in the healthy controls. A higher level of irisin in the plasma was associated with impaired overall cognition, specifically executive function. Linear regression analysis suggested that irisin (*p* = 0.017) and glycosylated hemoglobin (*p* = 0.036) were independent factors of diabetic MCI.

**Conclusions:** The level of irisin in the plasma correlated with cognitive impairment in T2DM patients, particularly with executive function. These results further suggest that, in addition to poor glycemic control, a high level of irisin in the plasma portends early cognitive deficits clinically in Chinese patients with T2DM.

## Introduction

Individuals with type 2 diabetes mellitus (T2DM) exhibit a higher prevalence of mild cognitive impairment (MCI) in comparison with the general population (1). The risk of cognitive impairment in DM patients is 1.2 to 1.5-fold higher than that reported in non-DM individuals (2). Cognitive impairment is considered one of the chronic complications of DM (3). Several potential mechanisms promote the occurrence of cognitive impairment in T2DM, including hyperglycemic toxicity, insulin resistance, oxidative stress, accumulation of amyloid-beta peptide and tau hyper-phosphorylation (4–9).

Irisin, a novel glycosylated polypeptide hormone, plays an important role in the homeostasis and metabolism of energy balance (10). It had been reported to lead to brown-fat-like development by stimulating the expression of uncoupling protein-1 and altering that of several molecules (11, 12). Irisin induces the transformation of white adipose tissue into brown adipose tissue. Consequently, the increased thermogenesis may lead to weight loss and improve insulin sensitivity and glucose tolerance in mice (13, 14). The increase of circulating irisin is associated with endurance training induced reduction of abdominal visceral fat in old and middle-aged people (15). Many studies have found low level of irisin in individuals with T2DM compared to that in non-diabetic individuals (16–19). Continuous exposure to hyperglycemia and impaired insulin signaling are major causes of Alzheimer's disease (AD) and related to cognitive impairment, especially learning and memory loss (20, 21). Several studies reported that irisin may regulate insulin resistance and glucose homeostasis (12, 22, 23), potentially improving cognitive function. Furthermore, irisin may promote neurogenesis (24) and protect against neuronal damage caused by oxidative stress (25, 26). In addition, it was shown that irisin regulates the production of brain-derived neurotrophic factor (27, 28), which may enhance cognitive function and reduce synaptic dysfunction in AD (29). These findings suggest that irisin may improve cognitive function.

Therefore, the aim of this study was to investigate the association between the level of irisin in the plasma and cognition performance in patients with T2DM.

## Materials and Methods

### Patients and Study Design

We recruited 133 patients (aged 45–75 years) who were admitted to the Department of Endocrinology of the Affiliated Zhongda Hospital of Southeast University (Nanjing, China) between February 2015 and June 2017. The patients were diagnosed with T2DM for ≥3 years according to the 1999 World Health Organization criteria (30). The exclusion criteria were as follows: (1) central nervous system diseases (i.e., recent stroke, head trauma, epilepsy, Parkinson's disease, depression, or other psychological illnesses) that may cause MCI; (2) drug or alcohol abuse or dependence; (3) other major illnesses, including cancer, anemia, or thyroid dysfunction; and (4) use of potential or known cognition-impairing drugs in the previous 3 months. All patients were of Chinese Han ethnic origin and provided written informed consent prior to their participation in the study. The study was approved by the Research Ethics Committee of the Affiliated Zhongda Hospital of Southeast University, Nanjing, China.

### Collection of Clinical Data

We collected the demographic and clinical characteristics of the patients, including gender, age, educational level, contact details, duration of T2DM, medical history (e.g., hypertension and fatty liver), fasting blood glucose, fasting C-peptide (FCP), glycosylated hemoglobin (HbA1c), triglyceride, total cholesterol, low-density lipoprotein, and high-density lipoprotein. We obtained physical measurements (i.e., weight, height, blood pressure, and waist and hip circumference) using a standard balance beam scale. The body mass index (BMI) is defined as the body weight (kg) divided by the square of the body height (m^2^).

### Measurement of the Level of Irisin in the Plasma

We collected blood samples in the morning after the patients lying down for a night, and instructed the patients to avoid intense physical activity the day before. The concentration of irisin in the plasma was measured using an enzyme-linked immunosorbent assay (ELISA) kit (Cusabio, Wuhan, China) according to the instructions provided by the manufacturer. The accuracy of this kit is comparable to that of EK-067-29 produced by Phoenix Pharmaceuticals, USA (31).

### Neuropsychological Testing

Neuropsychological testing, including the Montreal Cognitive Assessment (MoCA), Mini Mental State Exam (MMSE), digit span test (DST), verbal fluency test (VFT), clock drawing test, logical memory test (LMT), auditory verbal learning test, and trail making tests A and B (TMT-A and TMT-B) were performed to assess the cognitive functions (i.e., memory, attention, executive function, psychomotor speed, and visuospatial skills). Based on the MoCA scoring system, 59 patients with MoCA scores <26 and the remaining 74 patients with MoCA scores ≥26 were classified in the MCI group and normal cognition group (control group), respectively. One point was added to the MoCA score for patients with a number of education years <12 (32).

### Statistical Analysis

Statistical analyses were performed using the SPSS Version 21.0 software (IBM Corp., Armonk, NY, USA). For continuous variables, analysis of variance and Student's *t*-test were used to compare differences between groups at baseline. The chi-squared (χ^2^) test was employed for categorical variables. Spearman's correlation was used to examine the correlation between neuropsychological test scores and the level of irisin in the plasma. The relationship of cognitive performance with the level of irisin in the plasma, as well as demographic and clinical characteristics, was investigated using multiple linear regression analysis. A *p* < 0.05 denoted statistical significance.

## Results

### Demographic and Clinical Characteristics

Table 1 lists the baseline characteristics and neuropsychological test scores of the patients. There were no significant differences found between the MCI and control groups in terms of age, gender, educational level, prevalence of hypertension, duration of T2DM, history of alcohol abuse or dependence, smoking history, BMI, waist-to-hip ratio, fasting blood-glucose, 2-h plasma glucose, FCP, HbA1C, total cholesterol, triglyceride, low-density lipoprotein, high-density lipoprotein, apolipoprotein A1, and apolipoprotein B (*p* > 0.05). The MCI group demonstrated a significantly higher level of irisin in the plasma and homeostasis model of assessment for insulin resistance (HOMA-IR) than the control group (*p* < 0.05). Moreover, significant differences between the two groups were also observed in the neuropsychological test scores (*p* < 0.01). The memory, attention, executive function, psychomotor speed, and visuospatial skills in the MCI group were significantly lower compared with those reported in the control group.

**Table 1:** Demographic and clinical characteristics.

**Characteristic**	**MCI group (*n* = 59)**	**Control group (*n* = 74)**	***p*-value**
Age (y)	59.76 ± 6.887	58.3 ± 8.461	0.283
Male/Female, *n* (%)	31 (52.5)/28 (47.5)	46 (62.2)/28 (37.8)	0.264
Education level (y)	9 (9–12)	11 (9–12)	0.506
Duration of diabetes(y)	11.288 ± 5.7866	9.696 ± 5.3857	0.104
Hypertension *n* (%)	37 (62.7)	43 (58.1)	0.256
Hypertension duration (y)	5 (0–13)	4 (0–10)	0.508
Fatty liver *n* (%)	30 (50.8)	30 (40.5)	0.237
Smoking *n* (%)	20 (33.9)	28 (37.8)	0.640
Drinking *n* (%)	12 (20.3)	19 (25.7)	0.471
Systolic BP (mmHg)	135.25 ± 18.502	136 ± 15.67	0.802
Diastolic BP (mmHg)	81.29 ± 11.174	80.5 ± 9.77	0.665
FBG (mmol/L)	8.3578 ± 2.71327	7.7147 ± 2.50359	0.159
2h-PG (mmol/L)	15.0769 ± 3.57025	14.3576 ± 3.8885	0.274
HbA1C (%)	9.7186 ± 2.59698	8.9243 ± 2.16414	0.057
FCP	1.2446 ± 0.75122	0.854 (0.4418-1.603)	0.141
HOMA-IR	0.4385 ± 0.23212	0.2889 (0.1557–0.5094)	0.032
BMI (kg/m^2^)	25.12 ± 3.409	24.84 ± 3.045	0.903
WHR	0.9467 ± 0.06122	0.9366 ± 0.06408	0.355
TC (mmol/L)	4.7636 ± 1.08117	4.5538 ± 1.08846	0.372
TG (mmol/L)	1.8105 ± 1.14686	1.7174 ± 1.03957	0.625
LDL (mmol/L)	2.9275 ± 0.82897	2.8178 ± 0.80722	0.443
HDL (mmol/L)	1.1827 ± 0.33954	1.1749 ± 0.25705	0.88
ApoA1 (g/L)	1.0878 ± 0.26026	1.0732 ± 0.24375	0.74
ApoB (mmol/L)	0.82 ± 0.20130	0.8099 ± 0.18437	0.763
LPa (mmol/L)	258.23 ± 228.391	190.5 (108.75–310.75)	0.983
Irisin (ng/mL)	241.48 (145.208–564.815)	139.86 (82.845–500.413)	0.043
**Cognition test levels**			
MoCA	23 (20–24)	27 (26–28)	<0.001
VFT	14 (13–17)	16 (14–19)	<0.001
LMT	6.32 ± 4.22	10.09 ± 4.457	<0.001
DST	10.46 ± 2.054	12.03 ± 1.828	<0.001
CDT	3(2–4)	4 (3–4)	0.01
TMT-A	66 (53–85)	53 (45–58)	<0.001
TMT-B	196.44 ± 88.644	139.59 ± 48.465	<0.001
AVLT-immediate recall	15.78 ± 5.243	18.85 ± 3.788	<0.001
AVLT-delayed recall	5 (3–6)	6 (5–7)	<0.001

*Continuous variables were presented as mean ± SD, while categorical variables were presented as n (%) or median (interquartile range). Student's t-test was used to compare normally distributed variables between two groups. The Mann–Whitney U-test was used for the comparison of asymmetrically distributed variables. MCI, mild cognitive impairment; FBG, fasting blood-glucose; 2h-PG, 2-h plasma glucose; HbA1c, glycosylated hemoglobin; FCP, fasting C-peptide; HOMA-IR, homeostasis model of assessment for insulin resistance; BMI, body mass index; WHR, waist-to-hip ratio; TG, triglyceride; TC, total cholesterol; LDL, low density lipoprotein; HDL, high density lipoprotein; ApoA1, apolipoprotein A1; ApoB, apolipoprotein B; LPa, lipoprotein(a); MoCA, montreal cognitive assessment; VFT, verbal fluency test; LMT, logical memory test; DST, digit span test; CDT, clock drawing test; TMT-A, trail making test-A; TMT-B, trail making test-B; AVLT, auditory verbal learning test; SD, standard deviation*.

### Correlation of the Level of Irisin in the Plasma With Baseline Data and Cognitive Indicators

We subsequently explored the correlation of the level of irisin in the plasma with clinical and the different neuropsychological test scores in all patients using Spearman rank correlation analysis. The level of plasma irisin was positively correlated with the BMI, FCP, HOMA-IR, and TMT-A and TMT-B scores. Of note, it was negatively correlated with the MoCA, MMSE, VFT, LMT, and DST score in total (Table 2). The level of irisin was positively associated with the FCP and the TMT-A score, whereas it was negatively associated with the MoCA, MMSE, and VFT score in the MCI group. In healthy-cognition controls, the level of irisin was only positively correlated with the FCP and TMT-A score. Considering the relationship between irisin and BMI, we performed a hierarchical analysis. The level of irisin in the plasma was only positively correlated with the TMT-A score in patients with normal weight. In overweight patients, it was significantly positively correlated with the BMI, FCP, duration of T2DM, and TMT-A and TMT-B scores. In contrast, it was negatively correlated with the MoCA, MMSE, VFT, LMT, DST, and auditory verbal learning test-delayed scores (Table 2).

**Table 2:** Correlation of the level of irisin in the plasma with baseline data and cognitive indicators.

	**Total (*n* = 133)**	**MCI group (*n* = 59)**	**Control group (*n* = 74)**	**BMI ≤ 24 (*n* = 51)**	**BMI > 24 (*n* = 82)**
	***r***	***r***	***r***	***r***	***r***
BMI	0.217^*^	0.251	0.182	0.015	0.318^**^
WHR	0.023	−0.031	0.039	−0.129	0.059
FCP	0.268^**^	0.289^*^	0.237^*^	0.262	0.261^*^
HOMA-IR	0.217^*^	0.245	0.173	0.193	0.201
Duration of diabetes	0.165	0.104	0.167	0.055	0.224^*^
MoCA	−0.236^**^	−0.326^*^	−0.072	−0.169	−0.277^*^
MMSE	−0.234^**^	−0.305^*^	−0.001	−0.267	−0.241^*^
VFT	−0.248^**^	−0.394^**^	−0.092	−0.220	−0.258^*^
TMT-A	0.336^**^	0.295^*^	0.269^*^	0.323^*^	0.359^**^
TMT-B	0.237^**^	0.246	0.142	0.193	0.263^*^
LMT	−0.231^**^	−0.245	−0.125	−0.099	−0.346^**^
DST	−0.262^**^	−0.211	−0.204	−0.172	−0.334^**^
CDT	−0.072	−0.051	−0.018	−0.153	−0.015
AVLT-immediate	−0.107	−0.057	−0.046	−0.099	−0.109
AVLT-delayed	−0.149	−0.240	0.008	−0.028	−0.237^*^

*MCI, mild cognitive impairment; BMI, body mass index; WHR, waist-to-hip ratio; FCP, fasting C-peptide; HOMA-IR, homeostasis model of assessment for insulin resistance; MoCA, montreal cognitive assessment; MMSE, mini mental state examination; VFT, verbal fluency test; TMT-A, trail making test-A; TMT-B, trail making test-B; LMT, logical memory test; DST, digit span test; CDT, clock drawing test; AVLT, auditory verbal learning test*.

* p < 0.05 and

** *p < 0.01*.

### Correlation of the MoCA Score With Baseline Characteristics in All Patients

The relationship between the MoCA score and baseline characteristics was assessed through Spearman rank correlation analysis. We found that the MoCA score correlated with sex, irisin, educational level, HbA1c, FCP, and HOMA-IR (Table 3). Subsequently, we formed a multiple linear regression model to identify independent factors associated with MCI. Age, sex, irisin, educational level, duration of T2DM, HbA1C, FCP, waist circumference, BMI, waist-to-hip ratio, triglyceride, total cholesterol, high-density lipoprotein, low-density lipoprotein, and HOMA-IR were entered as independent variables in the multiple step-wise linear regression analysis, with the MoCA score as the dependent variable. The multivariable regression analysis revealed that plasma irisin, educational level, and HbA1c were associated with MCI in T2DM patients (*p* < 0.05) (Table 4). These three independent variables explained 17% of the variance.

**Table 3:** Correlation of the MoCA score and baseline characteristics.

	**Total (*****n*** **=** **133)**		**MCI group (*****n*** **=** **59)**		**Control group (*****n*** **=** **74)**	
	***r***	***p*-value**	***r***	***p*-value**	***r***	***p*-value**
Sex	−0.189	0.029	−0.373	0.004	−0.092	0.435
BMI	−0.013	0.883	−0.344	0.008	0.175	0.136
WC	−0.087	0.319	−0.298	0.022	0.208	0.075
Educational level	0.221	0.010	0.543	<0.001	0.162	0.167
Duration of diabetes	−0.137	0.116	−0.115	0.424	−0.154	0.168
Irisin	−0.236	0.006	−0.326	0.012	−0.072	0.540
HbA1c	−0.173	0.046	−0.146	0.271	0.177	0.131
FCP	−0.208	0.016	−0.158	0.231	−0.226	0.053
HOMA-IR	−0.239	0.006	−0.194	0.141	−0.149	0.205

*MCI, mild cognitive impairment; BMI, body mass index; WC, waist circumference; HbA1c, glycosylated hemoglobin; FCP, fasting C-peptide; HOMA-IR, homeostasis model of assessment for insulin resistance*.

**Table 4:** Multivariable linear regression analysis with the MoCA score as the dependent variable.

	**B**	**SE**	**Sig**.	**95% CI**
Irisin	−0.002	0.001	0.017	−0.004–0
Educational level	0.334	0.113	0.004	0.110–0.559
HbA1c	−0.261	0.123	0.036	−0.504 to −0.018

*Independent variables entered included: sex, age, irisin, educational level, duration of diabetes, HbA1C, FCP, WC, BMI, WHR, TG, TC, HDL, LDL, and HOMA-IR. MoCA, montreal cognitive assessment; HbA1c, glycosylated hemoglobin; T2DM, type 2 diabetes mellitus; FCP, fasting C-peptide; WC, waist circumference; BMI, body mass index; WHR, waist-to-hip ratio; TG, triglyceride; TC, total cholesterol; LDL, low-density lipoprotein; HDL, high-density lipoprotein; HOMA-IR, homeostasis model of assessment for insulin resistance; B, regression coefficients; SE, standard error; CI, confidence interval*.

## Discussion

The results of the present study demonstrated that the level of irisin in the plasma of T2DM patients with MCI increased. This level was negatively associated with the MoCA, MMSE, VFT, LMT, and DST scores, whereas it was positively correlated with the TMT-A and TMT-B scores. These findings indicated worse overall cognitive, executive, and attention functions. The multivariable regression analysis indicated that a high level of plasma irisin and HbA1c may play important roles in the development of MCI in T2DM patients.

This was the first study to investigate the correlation between the level of irisin in the plasma and cognitive function in T2DM patients. Correlations were found between irisin and the MoCA, MMSE, VFT, LMT, DST, TMT-A, and TMT-B scores. Higher levels of irisin indicated poorer cognitive function in the patients. Additionally, we found that the BMI, FCP, and HOMA-IR were positively correlated with irisin, suggesting that the increased level of irisin in T2DM patients may be correlated with higher BMI, FCP, and HOMA-IR values. Previous studies suggested that the level of plasma irisin was increased in obese subjects (33–35). Furthermore, several studies showed a significant association between irisin and the HOMA-IR index (35–38), which reflects insulin resistance. All aforementioned factors have been shown to promote the development of cognitive impairment in T2DM patients (39, 40). Thus, an increased level of irisin may be a predictive factor of T2DM-associated cognitive impairment.

Based on these findings, the relationship between irisin and cognitive function is contrary to that reported by Lourenco et al. (41), which suggested that a lower level of irisin correlated with cognitive impairment. Irisin was reduced in the hippocampi and cerebrospinal fluid of AD patients compared with MCI patients or cognitively normal individuals (41). However, the level of irisin in the plasma was not significantly different in AD compared with that measured in non-demented controls. Another study suggested that irisin was positively associated with overall cognition and memory (42). Moreover, rat models showed that treatment with irisin reduced neurological deficits (25, 43) and increased hippocampal synaptic plasticity (41). The difference between our study and the others was the study population. T2DM is often accompanied by metabolic dysfunction. Similar to the increased level of insulin in insulin resistance (37, 44), the level of irisin in the plasma increased in MCI patients. In the present study, the positive association between the level of irisin in the plasma and markers of insulin resistance in T2DM patients may demonstrate an adaptive response to obesity through irisin (18). Similarly, an increased level of irisin in MCI patients may be the result of irisin resistance. This notion was supported by the negative correlation between the level of irisin and MoCA score in the MCI group rather than the control group. Considering the positive correlation between irisin and the BMI, irisin is likely to be involved in inflammatory and oxidative stress (45, 46).

In addition, in this study, the MoCA score was negatively correlated with HbA1c and positively correlated with the educational level. These findings were congruous with those reported by previous studies. Chronic exposure to hyperglycemia may damage cognitive function (47, 48). Higher education and more thinking may delay the progression of dementia (49, 50). Consequently, we encourage the elderly to participate in more intellectual activities to delay the impairment of cognitive function.

Several limitations of this study should be considered. Firstly, it was a cross-sectional study with a small sample size, which may limit the robustness of the results. Secondly, there were concerns regarding the accuracy of the irisin antibody kits (51). Further validation analyses using mass spectrometry are required to address these concerns. Thirdly, most of the hospitalized patients had uncontrolled diabetes, which lead to the selection bias in the sample. Although we have carefully considered and implemented the inclusion and exclusion criteria in order to reduce bias, it still affected the results. Moreover, we only used the MoCA for MCI diagnosis in our diabetic patients. Finally, the study did not collect data regarding the intensity of daily exercise, which may play an important role in the level of irisin and MCI.

## Conclusion

In summary, this study showed that the MCI group had a higher level of irisin vs. the control group. Additionally, a higher level of irisin was associated with overall cognitive impairment, especially poorer executive function. Furthermore, irisin, HbA1c, and the educational level were identified as independent variables of MCI in all individuals, suggesting that low levels of irisin and HbA1c may be good predictors of MCI in T2DM patients. Further evidence, especially from longitudinal studies, is required to investigate the value of irisin as a predictive biomarker of MCI in T2DM patients.

## Data Availability

The datasets generated for this study are available on request to the corresponding author.

## Ethics Statement

The studies involving human participants were reviewed and approved by the Research Ethics Committee of the Affiliated Zhongda Hospital of Southeast University. The patients/participants provided their written informed consent to participate in this study.

## Author Contributions

SW, HL, and YY contributed to the conception and design of the study. HL conducted the study and statistical analysis, and wrote the manuscript. ST participated in the data analysis and interpretation. JH, RH, DG, JW, and KA performed the data collection. All authors read and approved the submitted version of this manuscript.

### Conflict of Interest Statement

The authors declare that the research was conducted in the absence of any commercial or financial relationships that could be construed as a potential conflict of interest.
